# Comprehensive Survey of Domiciliary Triatomine Species Capable of Transmitting Chagas Disease in Southern Ecuador

**DOI:** 10.1371/journal.pntd.0004142

**Published:** 2015-10-06

**Authors:** Mario J. Grijalva, Anita G. Villacis, Sofia Ocaña-Mayorga, Cesar A. Yumiseva, Ana L. Moncayo, Esteban G. Baus

**Affiliations:** 1 Tropical Disease Institute, Department of Biomedical Sciences, Heritage College of Osteopathic Medicine, Ohio University, Athens, Ohio, United States of America; 2 Center for Infectious and Chronic Disease Research, School of Biological Sciences, Pontifical Catholic University of Ecuador, Quito, Ecuador; Universidad Autónoma de Yucatán, MEXICO

## Abstract

**Background:**

Chagas disease is endemic to the southern Andean region of Ecuador, an area with one of the highest poverty rates in the country. However, few studies have looked into the epidemiology, vectors and transmission risks in this region. In this study we describe the triatomine household infestation in Loja province, determine the rate of *Trypanosoma cruzi* infection in triatomines and study the risk factors associated with infestation.

**Methodology/Principal Findings:**

An entomological survey found four triatomine species (*Rhodnius ecuadoriensis*, *Triatoma carrioni*, *Panstrongylus chinai*, and *P*. *rufotuberculatus)* infesting domiciles in 68% of the 92 rural communities examined. Nine percent of domiciles were infested, and nymphs were observed in 80% of the infested domiciles. Triatomines were found in all ecological regions below 2,200 masl. We found *R*. *ecuadoriensis* (275 to 1948 masl) and *T*. *carrioni* (831 to 2242 masl) mostly in bedrooms within the domicile, and they were abundant in chicken coops near the domicile. Established colonies of *P*. *chinai* (175 to 2003 masl) and *P*. *rufotuberculatus* (404 to 1613 masl) also were found in the domicile. Triatomine infestation was associated with surrogate poverty indicators, such as poor sanitary infrastructure (lack of latrine/toilet [w = 0.95], sewage to environment [*w* = 1.0]). Vegetation type was a determinant of infestation [*w* = 1.0] and vector control program insecticide spraying was a protective factor [*w* = 1.0]. Of the 754 triatomines analyzed, 11% were infected with *Trypanosoma cruzi* and 2% were infected with *T*. *rangeli*.

**Conclusions/Significance:**

To date, only limited vector control efforts have been implemented. Together with recent reports of widespread sylvatic triatomine infestation and frequent post-intervention reinfestation, these results show that an estimated 100,000 people living in rural areas of southern Ecuador are at high risk for *T*. *cruzi* infection. Therefore, there is a need for a systematic, sustained, and monitored vector control intervention that is coupled with improvement of socio-economic conditions.

## Author Summary

Chagas disease is a major health problem in Latin America. Its infectious agent, *Trypanosoma cruzi*, is transmitted mainly by the feces of insects called triatomines. Although great progress has been made in reducing transmission in the countries in the southern cone of South America, limited progress has been made in the Andean countries, partly because of lack of knowledge and partly because of limited resources and technical capability of control efforts. We report the first entomological survey conducted in southern Ecuador to learn the distribution of triatomines, their habitat and microhabitat preferences, and factors that favor their presence. The results of the study show that 9% of households in 92 rural communities harbor well-established triatomine populations. These rural areas in the southern Andean region are among the poorest in the country; most of the population lives in sub-standard housing, under conditions that favor the presence of Chagas disease vectors. These results, combined with recent reports of widespread triatomine infestation of wild animal nests in sylvatic areas throughout the region and frequent reports of reinfestation after control intervention, indicate the need for effective, monitored, and sustained vector control to reduce the high risk of infection to which approximately 100,000 people living in rural areas of southern Ecuador are exposed.

## Introduction

Chagas disease constitutes a serious problem in Latin America, where it has been estimated that about 10 million people are infected with the causative parasite, *Trypanosoma cruzi* [1]. Important advances have been made in reducing transmission by the vector *Triatoma infestans*, particularly in countries within the southern cone of South America [1], [2], [3], and *Rhodinus prolixus* in Central America [4],[5]. However, limited progress has been achieved in Ecuador, where neither accurate estimations of prevalence nor of people at risk of becoming infected by *T*. *cruzi* currently exist.

In Ecuador, there is limited understanding of Chagas disease incidence and prevalence, and vector distribution and biology. Loja Province, located in the southern portion of the Andean region, has long been considered endemic for Chagas disease, but few studies had been focused there. However, some recent efforts have aimed to fill knowledge gaps about this region. For example, previously, little was known about the extent to which Chagas is a problem; now studies have found that 35% of households in some areas of Loja are infested with triatomines and cited seroprevalences of 3.6% and 3.9% [6], [7]. However, the sample size and geographical coverage of these serological studies prevent them from being representative of the entire region. In addition, there is ample documentation of the presence of *T*. *cruzi infected* sylvatic triatomine populations in this region [8].

Loja Province encompasses about 11,100 km^2^ characterized by a mix of hilly and mountainous topography that ranges from 150 to 3,800 meters above sea level (masl). This province has approximately 448,966 inhabitants, of which 58% live in rural areas. The main economic activity in this region is agriculture, especially coffee *(Coffea arabica)*, corn *(Zea mays)*, peanut *(Arachis hypogaea)*, and tree fruit. Poverty, defined as “unmet basic needs,” has been estimated at 60.1% for Ecuador overall and 83.4% for rural areas. In Loja, the prevalence of rural poverty is estimated to be 93.2% (all classified as chronic poverty). Indeed, several counties within this province exhibit the highest poverty rates in the country [9–11]

In Ecuador, 16 species of triatomines have been reported [12]. Studies in Loja province have documented a high prevalence of household infestation and colonization mainly by *Rhodnius ecuadoriensis* and *Triatoma carrioni*, and to a lesser extent household infestation without colonization by *Panstrongylus chinai*, *P*. *rufotuberculatus*, and *Eratyrus mucronatus* [6]. In addition, sylvatic *R*. *ecuadoriensis* highly infected with *T*. *cruzi* were recently reported in this region in association with squirrel nests [8,13].

This report presents the results of a province-wide survey designed to document the distribution of domiciliary triatomine infestation in Loja Province, describe the microhabitats where triatomines are found, determine the rate of trypanosome infection of triatomines, and study the risk factors associated with household infestation.

## Methods

### Study area

**Fig 1:**
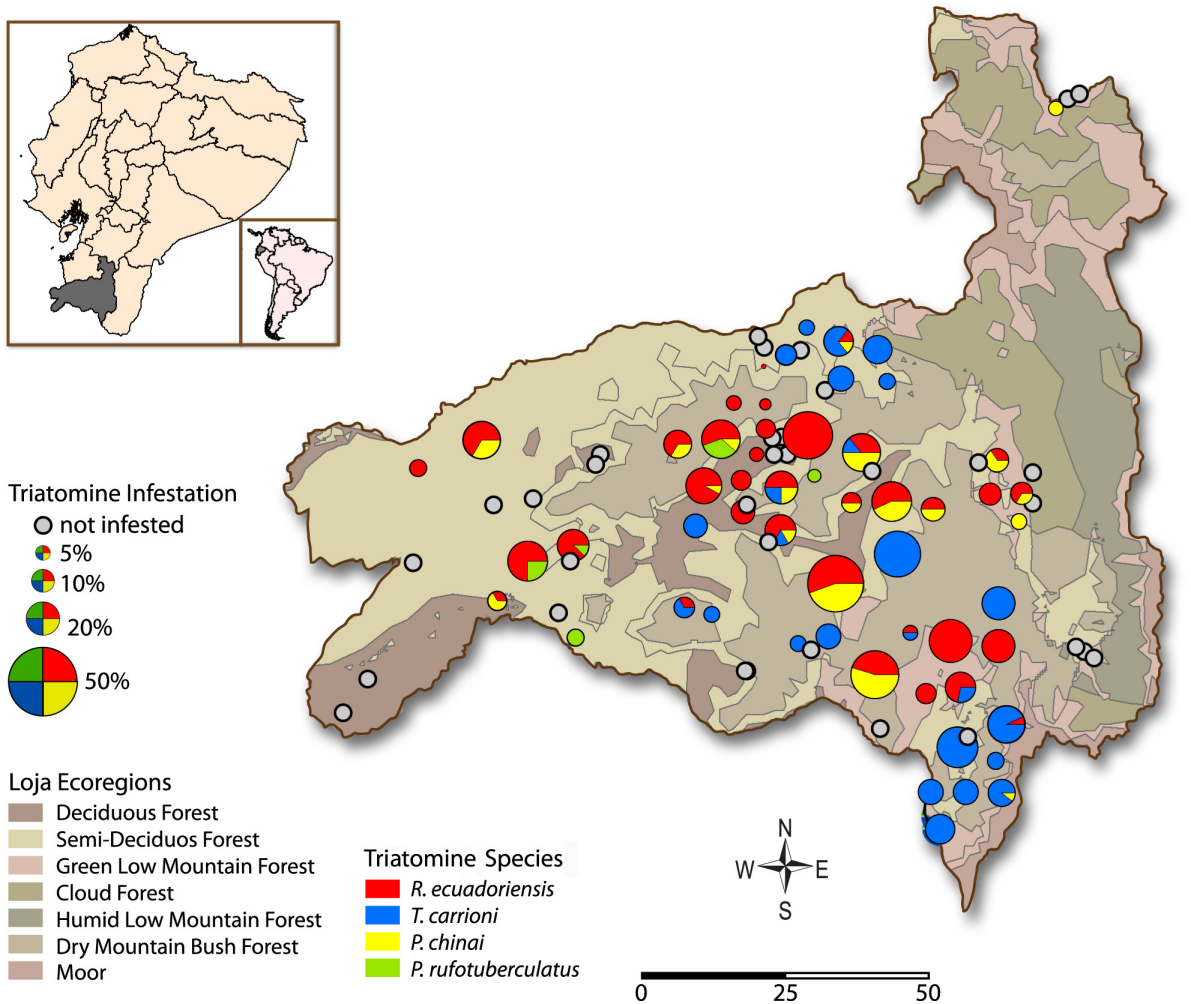
Triatomine infestation in Loja Province, a region of southern Ecuador having several ecological regions. Location of each community surveyed is marked. The size of the symbol corresponds to the % of houses infested with triatomines in each community. The colors represent the four triatomine species found: Red = *R*. *ecuadoriensis*, blue = *T*. *carrioni*, green = *P*. *rufotuberculatus* and yellow = *P*. *chinai*. Classification of ecological regions as per Sierra et al [14].

We visited 92 rural communities in all 16 counties of Loja Province between 2005 and 2009 (Fig 1). Community selection was based on a combination of a geographical location and accessibility. The sampled domiciles were at an altitude ranging from 150 to 2440 masl and included five vegetation zones: deciduous forest, semi-deciduous forest, green low mountain forest, cloud forest, and dry mountain bush forest [14].

### Ethics statement

Written informed consent was obtained from the head of the household following protocols approved by the institutional review boards of Ohio University and Pontifical Catholic University of Ecuador, and triatomines were collected under Ecuadorian collection permit N° 002–07 IC-FAU-DNBAPVS/MA.

### Household surveys

We visited all domiciles in each community (n = 4,782). The location of each domicile was recorded using GPS receivers (Garmin, Etrex), and photos were taken of the four sides of each domicile. We documented house construction materials and characteristics of the peridomestic area. A questionnaire was administered to the head of the household that included questions about livestock and insecticide spraying, among others. In 2008, the questionnaire was abridged to make it compatible with a common questionnaire being prepared by the Surveillance and Information Systems workgroup for use in endemic countries as part of the Chagas Disease initiative created by the Neglected Diseases Division of the World Health Organization (www.who.int).

### Community education

We delivered a 10-min educational talk about triatomines and Chagas disease at each house (3,191 DUs), supported by a booklet for each family member. The booklet contained color life-size pictures of adults and nymphs of local triatomine species, cartoon- and text-based explanations about triatomine feeding, *T*. *cruzi* transmission via feces, drawings of favored triatomine habitats in the intra and peridomicile, games (search the triatomine labyrinth, triatomine color, cut and paste, draw activities and word puzzle), instructions on how to safely collect and report triatomine infestation to the local health promoter, information about Chagas disease signs and symptoms, and practical recommendations to prevent triatomine infestation. A calendar (passive detection device) with educational information was affixed to one of the bedroom walls, usually near the bed. Educational talks, reinforced by presentation of a video, participatory classroom activities, and outdoor didactic games were conducted at the elementary schools in each community. This study does not include the evaluation of the effectiveness of these activities.

### Triatomine collection

Two-person teams searched domiciles and peridomiciles during one hour (30 minutes in each area) using a modification of the one-man-hour method previously described [6,15]. Briefly, if no bugs were found after the initial 20-min search, the searches were continued for an additional 10 min with the use of 6% aqueous pyrethrin solution (PRENTOX, ExciteR, Prentiss Inc., Sandesville, GA, USA). This solution was hand-sprayed in cracks and crevices as an irritant to prompt the exit of triatomines and increase collection efficiency [16]. Collected triatomines were placed in individually labeled plastic containers and transported to either the field laboratory or the insectary of the Center for Infectious and Chronic Disease Research at Pontifical Catholic University of Ecuador in Quito. Details of the insects’ place of capture (intra- or peridomicile and microhabitat), species, number of insects found dead or alive, and insects’ developmental stages were noted by each team in the field and corroborated by trained entomologists at the field laboratory [17], [18]. If live triatomines were found in or around the domicile, both environments were sprayed. Kitchen wares, food, bedding, clothes, and personal items were removed from infested domiciles before complete indoor and outdoor spraying of all surfaces of the dwellings and peridomestic structures. Spraying was done with 5% deltamethrin WP that was applied at 25 mg/m^2^ by trained personnel from the National Chagas Control program using Hudson X-pert sprayers (H. D. Hudson Manufacturing Co., Houston, TX). We instructed inhabitants to leave bedding and clothes exposed to direct sunlight and not to enter the dwelling for at least one hour after the spraying. At the end of the day, each team stored the remaining insecticide and the water used to wash the spraying equipment in a plastic container for use the following day [15].

### Natural infection with trypanosomes

Intestinal content of live triatomines was analyzed for the presence of *Trypanosoma sp*. by microscopy and PCR as previously described [8]. This procedure allows for discrimination between *T*. *cruzi* and *T*. *rangeli* and each PCR reaction was run with a positive control for *T*. *cruzi*, *T*. *rangeli* and a reagent control (without DNA). The infection index (100 x number of infected individuals/total number of analysed individuals) was calculated for each type of microhabitat. *Trypanosoma cruzi* discrete typing units (DTU) were characterized as previously described [19]. The sensibility and specificity (95% CI) of microscopy for detecting *Trypanosoma sp*. (*T*. *cruzi and/or T*. *rangeli*) infection in triatomines were calculated using PCR as the gold standard. The level of agreement between the two techniques was estimated by the Kappa index (k).

### Data management

House surveys were entered into a Microsoft Access database (Office 2003) using custom made data entry forms and the data was then exported to Excel (Office 2003). Entomological field and laboratory results were entered into Excel spreadsheets. Datasets were manually and independently compared twice with the source documents to ensure accuracy. Datasets were imported and merged into Statistical Package for the Social Sciences v17.0 (SPSS, Inc., Chicago, IL). Frequencies were calculated to identify outliers or response inconsistencies. Discrepancies related to house characteristics were resolved by examination of digital pictures taken of the domicile and peridomicile at every DU. Discrepancies that could not be resolved due to illegible hand writing in the forms or other obvious errors were eliminated from the data sets and reported as missing values.

### Data analysis

The distribution of each triatomine species was described in terms of the ecological zones and altitude where they were found. We also noted where several species overlapped. The following entomological indexes were calculated for only live triatomines: Infestation rate (100 x number of houses infested /number of houses searched), density (number of triatomines captured/number of houses searched), crowding (number of triatomines captured/number of houses infested), and colonization index (100 x number of houses with nymphs/number of houses infested) [20].

We conducted a descriptive analysis of the variables in the household surveys to characterize the domiciles and the extent of the infestation.

A multimodel inference approach based on Akaike’s Information Criterion (AIC) [21] was used to identify the strongest determinants for intradomicile and peridomicile infestation by *R*. *ecuadoriensis* and *T*. *carrioni*. First, for each outcome, we fit three subset of models: 1) only-household level covariates (roof, floor and wall materials, presence of toilet, type of sewage and water, crowding, cooking fuel, self-reported and vector control program insecticide spraying and intradomicile storage of firewood and agriculture products); 2) only domestic animals covariates (dogs, cats, guinea pigs, pigs and sheep/goats); 3) only peridomicile covariates (vegetation: bushes, brush/scrub and fruit trees and accumulation of: firewood, wool for construction, rock/brick piles, household trash, agriculture refuse and products). Unfortunately, important variables such as: roof and floor material construction, cooking fuel, type of sewage, sheep/goats were not included in the model for peridomicile infestation by *T*. *carrioni* because zero or small numbers in some categories. For the same reason, presence of chickens were not included in models for any bugs. Second, among each subset the models we selected variables according their relative importance (*w*>0.35) for predicting DUs infestation in order to fit the final models. Vegetation type variable was included in all final models. Final models considering every possible combination of selected variables were run: 12 variables gave 4096 models for intradomicile infestation by *R*. *ecuadoriensis*, 13 variables gave 8192 models for peridomicile infestation by *R*. *ecuadoriensis* and intradomicile infestation for *T*. *carrioni* and 9 variables gave 512 models for peridomicile infestation by *T*. *carrioni*.

The Akaike’s weight (*w*_*i*_) of each model was calculated as the quotient of the log-likehood of the particular model divided by the total sum of the log-likehood of all considered models. The relative importance of a particular variable (*w*) was then calculated as the sum of Akaike weights of all models that contained this particular variable. Variables with *w*>0.9 were considered of high importance in defining DUs infestation, variables with 0.7<*w*<0.9 were considered of secondary importance and variables with *w*<0.7 had limited contributions.

Finally, we performed model averaging to estimate weighted mean effect-sizes estimates (OR, 95% CI) resulted from averaging the parameter value in each model where the variable was present weighted by the Akaike weight of the respective model.

Descriptive analysis was conducted using Statistical Package for the Social Sciences v17.0 (SPSS, Inc., Chicago, IL). Multimodel inference and model averaging were performed in the software R (v. 2.12.1).

## Results

### Distribution of triatomines by species, ecological region, and altitude

The most common species was *R*. *ecuadoriensis*, which was found in 59% of the 63 infested communities. All *R*. *ecuadoriensis*-infested communities were located in the central and western portions of Loja at altitudes ranging from 275 to 1,948 masl (Fig 1) and encompassing four ecological zones: deciduous forest (60% of communities examined were infested, n = 20), semi-deciduous forest (33%, n = 43), green low mountain forest (47%, n = 15), and dry mountain bush forest (33%, n = 12). The second most common species was *T*. *carrioni*, which was found in 41% of infested communities. *T*. *carrioni* infestation was dominant in the northern and southern areas of the province. Overall, this species was found infesting domiciles at altitudes ranging from 831 to 2,242 masl and encompassing three ecological zones: semi-deciduous forest (30%), green low mountain forest (27%), and dry mountain bush forest (75%). Although less abundant, *P*. *chinai* was found in 32% of infested communities at domiciles located in a wide range of altitude (175 to 2,003 masl) and four ecological zones: deciduous forest (35%), semi-deciduous forest (19%), green low mountain forest (27%), and dry mountain bush forest (8%). Finally, *P*. *rufotuberculatus* was found in 11% of the infested communities in domiciles located at altitudes ranging from 404 to 1,613 masl and in three ecological zones: deciduous forest (5%), semi-deciduous forest (12%), and green low mountain forest (7%). Thus, simultaneous infestation of species occurred in many communities: *R*. *ecuadoriensis* and *P*. *chinai* were both found in 24% of infested communities; *R*. *ecuadoriensis* was also found with *T*. *carrioni* in 13% of infested communities and with *P*. *rufotuberculatus* in 3%. *P*. *chinai* was found with *T*. *carrioni* in 6% of infested communities and with *P*. *rufotuberculatus* in 2% (Fig 1). Triatomine infestation was not found in the two communities located in the cloud forest ecological zone.

### Infestation indexes

Four species of triatomines, *R*. *ecuadoriensis*, *T*. *carrioni*, *P*. *chinai*, *and P*. *rufotuberculatus*, were found in or around 8.8% of the domiciles examined (S1 Table). A total of 11,115 live triatomines, 372 dead triatomines, and 614 triatomine eggs were collected. Only live triatomines were included in the analyses. The density was 3.5 insects per examined domicile, the crowding was 39 bugs per infested domicile, and the colonization index was 80% (S1 Table). Triatomines were found in 68% of the 92 communities searched. Twenty (32%) of the 63 infested communities had infestation rates between 10% and 20%, ten (16%) presented infestation rates between 20% and 30%, and 4 (6%) presented infestation rates higher than 30% (S1 Table). The highest infestation rate (48%) was found in Chaquizhca, Calvas County. The highest density (46.5 bugs per domicile searched) and crowding (270 bugs per infested domicile) were found in Tacoranga, Paltas county. A 100% colonization index was observed in 51% of the infested communities (32 of 63; S1 Table).

### Species-specific entomological indexes and habitat

*R*. *ecuadoriensis* was found infesting 4.1% of searched domiciles. This species showed a preference for the peridomestic habitat, where it was 11 times more abundant than in the domestic habitat (Table 1). Thus, infestation, colonization (presence of nymphs), density, and crowding by this species were higher in the peridomicile than in the domicile. *T*. *carrioni* was found in 3.4% of searched domiciles. Although 1.5 times more bugs were captured in the peridomicile than in the domicile, the number of houses with intradomiciliary infestation was 2.4 times higher than those with peridomiciliary infestation. Nevertheless, all entomological indexes were higher in the peridomicile. Conversely, the less abundant *P*. *chinai* and *P*. *rufotuberculatus* were found mostly in the intradomicile and had significantly lower colonization indexes (Table 1).

**Table 1:** Triatomine species found in domiciles and peridomiciliary areas of rural communities in Loja Province.

Species^a^	Entomological indexes^b^							Colonization (%)
	Live Triatomines collected	Infested domiciles	Domiciles with nymphs	Infestation (%)	Density	Crowding	Median (IR)^c^	
Intradomicile								
*R*. *ecuadoriensis*	700	67	47	2.1	0.2	10.4	3 (1–6)	70.1
*T*. *carrioni*	846	87	68	2.7	0.3	9.7	5 (2–10)	78.2
*P*. *chinai*	173	44	27	1.4	0.1	3.9	1 (3–5)	61.4
*P*. *rufotuberculatus*	47	10	6	0.3	0.0	4.7	2 (1–4)	60.0
Total	1,768	208	148	6.5	0.6	8.5	4 (2–9)	71.2
Peridomicile								
*R*. *ecuadoriensis*	7,762	79	73	2.5	2.4	98.3	26 (9–124)	92.4
*T*. *carrioni*	1,347	36	33	1.1	0.4	37.4	10.5 (4.3–31)	91.7
*P*. *chinai*	235	12	8	0.4	0.1	19.6	5 (2–15)	66.7
*P*. *rufotuberculatus*	5	2	1	0.1	0.0	2.5	2.5 (1–4)	50.0
Total	9,349	129	115	4.0	2.9	72.5	15 (4–70)	89.1
All habitats								
*R*. *ecuadoriensis*	8,462	132	109	4.1	2.7	64.1	12 (2–49)	82.6
*T*. *carrioni*	2,193	107	88	3.4	0.7	20.5	6 (2–17)	82.2
*P*. *chinai*	408	51	32	1.6	0.1	8.0	3 (1–8)	62.7
*P*. *rufotuberculatus*	52	12	7	0.4	0.0	4.3	2 (1–3.8)	58.3
Total	11,115	282	226	9.5	3.5	39.4	7 (2–18)	80.1

^a^ *R*, *Rhodnius; T*, *Triatoma; P*, *Panstrongylus*.

^b^ Infestation rate (100 x number of houses infested /number of houses searched), density (number of triatomines captured/number of houses searched), crowding (number of triatomines captured/number of houses infested), and colonization index (100 x number of houses with nymphs/number of houses infested) (WHO 2002).

^c^ Median and Interquartile range (IR) of number of triatomines found in infested domestic units.

### Species-specific microhabitat preferences

*R*. *ecuadoriensis* was found to have clear preference for chicken nests in the peridomicile (91% of insects collected). In the intradomicile, this species was found mostly in or near the bed (89%) and was less commonly associated with chicken nests (4%) and guinea pigs (3%) kept inside the house. Similarly, *T*. *carrioni* was found mostly associated with chicken nests (64%) in the peridomicile and to a lesser degree, was also found in guinea pig pens, dog houses, and piles of wood, bricks, and firewood (22%). In the domicile, this species was found primarily in the bedroom (86%), with some infestation also in indoor chicken nests and guinea pig pens (8%). The less abundant *P*. *chinai* was found mostly in microhabitats located in the bedroom (75%) and in indoor guinea pig pens (10%). Finally, *P*. *rufotuberculatus* was found mainly in the bedroom (82%).

### Species-specific population structure

**Fig 2:**
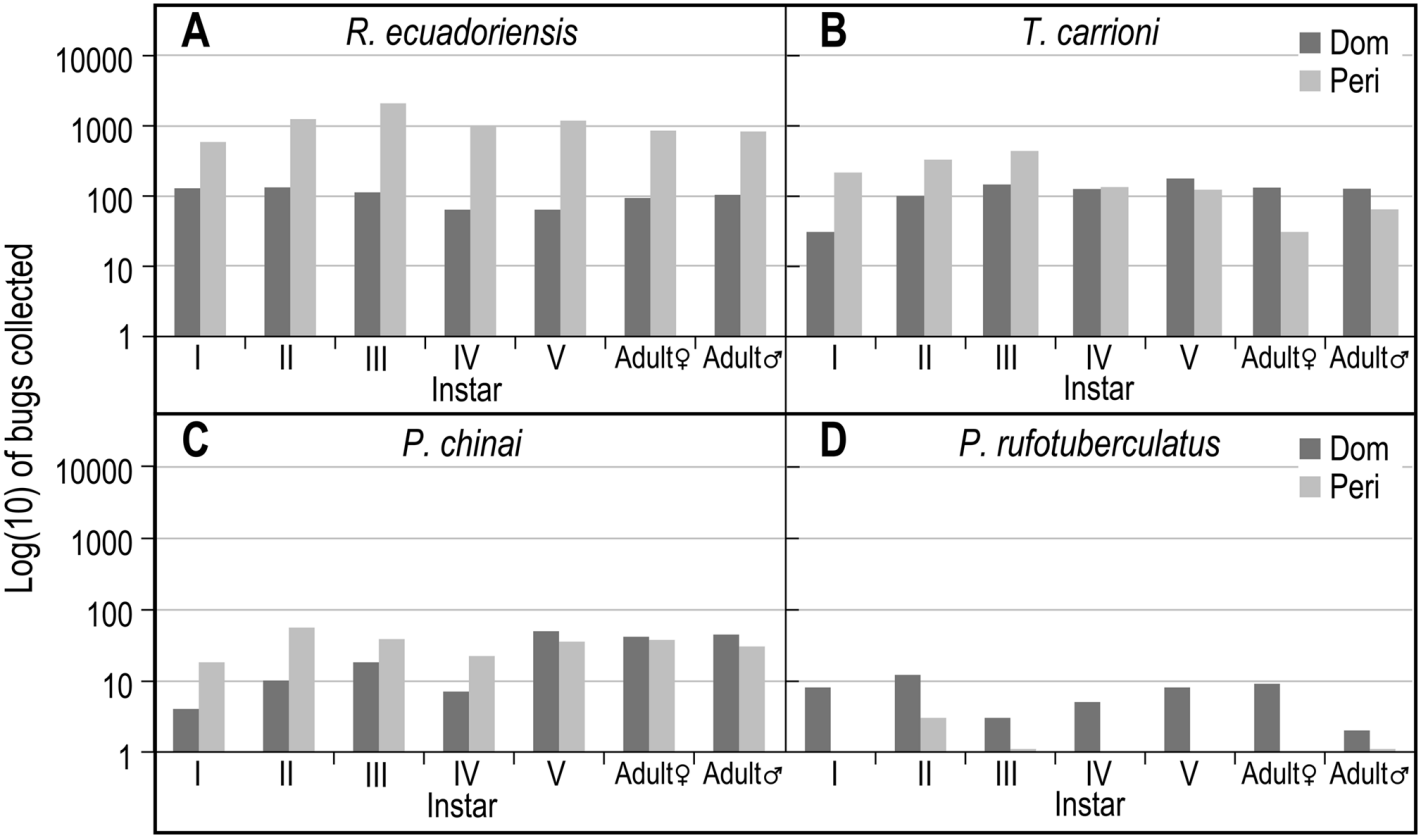
Population structure of triatomines collected in rural communities of Loja Province, Ecuador. Number of live Nymphal I—V instars and adult female and male bugs collected in domestic (Dom) and peridomestic (Peri) habitats. A) *R*. *ecuadoriensis*, B) *T*. *carrioni*, C) *P*. *chinai* and D) *P*. *rufotuberculatus*.

Fig 2 shows the population structure of each species collected. An abundance of *R*. *ecuadoriensis* and *T*. *carrioni* nymphal instars was found in and around domiciles, indicating that these were well established populations that were actively reproducing. Although with significantly fewer individuals, similar results were found for *P*. *chinai* in both domestic and peridomestic habitats and for *P*. *rufotuberculatus* in the domestic habitat. Males and females were found in similar proportions for all species within each habitat except for *P*. *rufotuberculatus*, for which more females were found.

### Characteristics of domiciles

The average population of each community was approximately 240 people. We visited 4,782 domiciles. Of these, 3,191 (67%) were examined for the presence of triatomines. The main reason for failing to search a house was that the house was closed or the head of the household was absent at the time of visit (66%). Other reasons were that the house was uninhabited (25%) or the head of the household refused to participate in this study (9%). House characteristics can be found in Table 2. Table 3 shows the characteristics of the peridomicile, including the presence or absence of accumulated materials and their distance from the domicile, and Table 4 shows the presence and median number of domestic animals and livestock. The vast majority of domiciles had chickens (91%), usually roosting in coops or pens located immediately adjacent to the domicile’s walls or between large roof eaves. Although dogs were common, only 5% of domiciles reported that dogs slept indoors. Cats were also common (57%) but often lived in a semi-feral condition. Finally, guinea pigs (*Cavia porcellus*) were kept by 25% of domiciles either for consumption or to be sold in the market. Most guinea pigs were kept either in pens adjacent to the domicile (83%) or roaming free within the house.

**Table 2:** Characteristic of domiciles from 92 communities located in rural areas of Loja Province.

House characteristic	n = 3,039^a^	%
**Roof material**		
Cement/asbestos/zinc	423	14%
Tile	2596	85%
Palm, other	20	1%
**Wall material**		
Cement/brick	785	26%
Adobe	2119	70%
Wood	43	1%
Cane, other	75	3%
**Floor**		
Cement/tile/wooden parquet	1001	33%
Wood boards	520	17%
Cane, other	20	1%
Dirt	1491	49%
**Size and inhabitants**		
Bedrooms ≤ 2	2276	76%
Inhabitant crowding ^b^ ^,^ ^c^	1124	53%
**Services**		
Electricity ^b^	1912	90%
No latrine	1246	41%
Public sewer system ^b^	119	6%
Sewage to environment ^b^	1279	60%
Piped water system ^b^	1324	62%
Septic tank	423	20%
Water from river or stream ^b^	669	32%
Sprayed < 12 months	464	15%
**Cooking fuel**		
Natural gas ^b^	1665	79%
Firewood/coal ^b^	1720	81%
**Intradomicile storage**		
Firewood ^b^	333	16%
Agricultural products ^b^	1262	60%

^a^ total n varies among each item from 2.996 to 3,039 due missing values.

^b^ total n varies among each item from 2,099 to 2,204 due to a change on the questionnaire form in 2008 and some missing values.

^c^ Inhabitant crowding = more than 3 people per bedroom.

**Table 3:** Peridomestic materials and vegetation, found in rural communities of Loja Province.

Item	Total^a^	n (%)	Distance in meters from domicile Median (IR)^b^
Firewood	2,097	1,354 (65)	3 (1–5)
Wood for construction	2,087	422 (20)	3 (1–5)
Rocks/brick piles	2,087	799 (38)	3 (1–5)
Household trash	2,085	1,265 (61)	6 (3–10)
Agricultural refuse	2,091	653 (31)	5 (2–20)
Agricultural products	2,089	534 (26)	3 (1–5)
Bushes (arbustos)	2,097	1,802 (86)	5 (3–10)
Fruit trees	2,088	1,215 (58)	5 (4–10)
Brush or scrub	2,090	1,671 (80)	5 (3–10)
Palm trees <30 m	2,078	83 (4)	-

^a^ total n varies among each item from 2,078 to 2,097 due to missing values.

^b^ Median and Interquartile Range of distance in meters from domicile (IR)

**Table 4:** Livestock found in rural communities of Loja Province.

Livestock	Total^a^	n (%)	Animals per domicile Median (IR)^b^
Chickens, other birds	3,029	2,740 (91)	10 (6–20)
Dogs	3,032	2,442 (81)	2 (1–3)
Guinea pigs (outdoor)	3,028	759 (25)	6 (4–10)
Pigs	3,031	1,994 (64)	3 (1–5)
Sheep or goats	3,029	588 (19)	5 (3–10)
Cats	3,015	1,716 (57)	1 (1–2)

^a^ total n varies among each item from 3,015 to 3,032 due to missing values.

^b^ Median and Interquartile Range (IR) of animals per domicile

### Risk factors for triatomine infestation

Data for factors associated with triatomine infestation in bivariate analysis are provided in S2 Table. The Akaike weight of each variable in the average model indicated that three variables could be considered of high importance in defining intradomicile infestation by *R*. *ecuadoriensis* with *w* > 0.9, one additional variable were of secondary importance with 0.7 < *w* < 0.9, and the remaining had limited contributions (Table 5). Houses with presence of pigs showed a high risk of infestation and had a very high weight (*w* = 1.0). Similarly, vegetation type was a very important variable (*w* = 1.0). Houses located in the green low mountain forest had a higher risk to be infested. Number of bedrooms >2 was a major protective factor (*w* = 1.0). A risk factor of secondary importance was keeping sheep/goats in peridomicile (*w* = 0.76)–it result in a 1.9 fold higher risk of infestation by *R*. *ecuadoriensis*. Finally, the lack of latrine/toilet (*w* = 0.67), the storage of agricultural products (*w* = 0.59) inside house and the presence of fruit trees (*w* = 0.68) and brush/scrub (*w* = 0.54) in the peridomicile had very minor weights in the model and may thus be of limited relevance as determinants for *R*. *ecuadoriensis* intradomicile infestation.

**Table 5:** Important determinants for intradomicile and peridomicile infestation by R. ecuadoriensis in Loja province (N = 2120).

	Intradomicile				Peridomicile			
Factor	Infested DUs	OR*	95% CI	RI	Infested DUs	OR**	95% CI	RI
	n (%)				n (%)			
**Presence of pigs**	40 (2.84)	3.29	0.88–12.32	1.0	51 (3.63)	2.79	0.81–9.63	1.0
**No. Bedrooms (>2)**	5 (0.94)	0.32	0.08–1.27	1.0				
**Vegetation Type**				1.0				1.0
Dry mountain forest	1 (0.34)	1.00			2 (0.69)	1.00		
Green_low mountain forest	19 (4.34)	13.22	1.79–97.84		16 (3.65)	5.95	1.06–33.40	
Semi_decidous forest	12 (1.38)	4.47	0.60–33.26		24 (2.75)	4.47	0.82–24.41	
Decidous forest	14 (3.06)	7.89	1.06–58.80		17 (3.71)	6.05	1.08–33.96	
Cloud forest	0 (0.0)	----	----		0 (0)	----	----	
**Presence of sheeps or goats**	18 (4.01)	1.87	0.60–5.82	0.76	19 (4.23)	1.44	0.48–4.31	0.37
**Fruit trees (peridomicile)**	19 (1.52)	0.58	0.19–1.77	0.68				
**Lack of latrine or toilet**	31 (3.37)	1.83	0.57–5.94	0.67	38 (4.13)	2.09	0.70–6.27	0.95
**Agricultural products (intradomicile)**	35 (2.73)	1.74	0.54–5.63	0.59	43 (3.35)	1.43	0.48–4.24	0.39
**Brush/scrub (peridomicile)**	40 (2.35)	1.90	0.51–7.11	0.54				
**Tile roof**	43 (2.38)	1.88	0.41–8.73	0.35				
**Self reported insecticide spraying**	21 (2.77)	1.40	0.47–4.16	0.32	31 (4.10)	2.08	0.74–5.84	1.0
**Agricultural refuse (peridomicile)**	19 (2.91)	1.38	0.45–4.20	0.30				
**Well drinking water**	21 (1.59)	0.76	0.24–2.42	0.25				
**VCP insecticide spraying**					10 (1.46)	0.29	0.09–0.99	1.0
**Presence of guinea pigs**					8 (1.59)	0.40	0.11–1.39	0.99
**Agricultural products (peridomicile)**					25 (4.41)	1.80	0.63–5.12	0.97
**Firewood (peridomicile)**					46 (3.34)	1.78	0.58–5.48	0.72
**Dirt floor**					38 (3.60)	1.66	0.56–4.88	0.64
**Presence of Dogs**					54 (3.10)	1.71	0.43–6.79	0.38
**Rocks/bricks (peridomicile)**					19 (2.38)	0.89	0.31–2.61	0.20

*Model_averaged effect-sizes (OR) from the final 4096-model set

**Model_averaged effect-sizes (OR) from the final 8192-model set

Relative importance (RI) of variables was assessed by multi-model inference based on Akaike's information criterion

OR: Odds Ratio; 95% CI: Confidence Interval

VCP: Vector Control Program

Important factors that increased the risk of peridomicile infestation by R. ecuadoriensis were: presence of pigs (*w* = 1.0), vegetation type (*w* = 1.0), self-reported insecticide spraying (*w* = 1.0), accumulation of agricultural products in the peridomicile (*w* = 0.97) and lack of latrine or toilet (*w* = 0.95) (Table 5). Vector control program insecticide spraying (*w* = 1.0) and presence of guinea pigs (*w* = 0.99) had a negative association with peridomicile infestation. Accumulation of firewood in the peridomicile evidenced a moderately high relative importance (*w* = 0.72) and a positive association with peridomicile infestation (Table 5).

The most relevant factors positively associated with *T*. *carrioni* intradomicile infestation (Table 6) were: sewage to environment (*w* = 1.0), keeping guinea pigs (*w* = 0.99) and presence of fruit trees in the peridomicile (*w* = 1.0). Vegetation type was also a high importance variable in defining infestation (*w* = 1.0). In addition, households located in the dry mountain forest and green low mountain forest result in 2.05 and 1.67 higher risk of infestation, respectively, when compared to households located in semi deciduous forest (reference category). A secondary predictor that increase the risk of infestation were dirt floor (*w* = 0.77) whereas well drinking water decrease the risk (*w* = 0.73). Other less important factors were: firewood cooking fuel (*w* = 0.55), presence of pigs (*w* = 0.52), self-reporting insecticide spraying (*w* = 0.46), presence of sheep/goats (*w* = 0.42), number of bedrooms >2 (*w* = 0.39) and accumulation of household trash in the peridomicile (*w* = 0.36).

**Table 6:** Important determinants for intradomicile and peridomicile infestation by *T*. *carrion*i in Loja province (N = 2120).

	Intradomicile				Peridomicile			
Factor	Infested DUs	OR*	95% CI	RI	Infested DUs	OR**	95% CI	RI
	n (%)				n (%)			
**Sewage to environment**	45 (3.51)	4.99	1.17–21.18	1.0				
**Vegetation Type**				1.0				1.0
Semi deciduous forest	15 (1.72)	1.00			4 (0.46)	1.37	0.17–10.98	
Green low mountain forest	21 (4.79)	1.67	0.51–5.53		8 (1.83)	5.36	0.70–41.13	
Dry mountain forest	13 (4.48)	2.05	0.58–7.17		1 (0.34)	1.00	0.17–10.98	
Deciduous forest	0 (0.0)	----	----		0 (0.0)	----	----	
Cloud forest	0 (0.0)	----	----		0 (0.0)	----	----	
**Presence of guinea pigs**	23 (4.56)	2.21	0.74–6.62	0.99				
**Fruit trees (peridomicile)**	37 (2.96)	2.17	0.69–6.87	0.99	10 (0.80)	2.03	0.41–10.11	0.37
**Dirt floor**	37 (3.51)	1.98	0.60–6.52	0.77				
**Well drinking water**	18 (1.36)	0.54	0.17–1.67	0.73	4 (0.30)	0.42	0.09–2.00	0.52
**Firewood/coal (cooking fuel)**	47 (2.73)	2.67	0.49–14.47	0.55				
**Presence of pigs**	38 (2.70)	1.67	0.51–5.44	0.52	7 (0.50)	0.60	0.13–2.66	0.30
**Self-reported insecticide spraying**	12 (1.59)	0.63	0.20–2.01	0.46	2 (0.26)	0.36	0.06–2.04	0.51
**Presence of sheeps or goats**	6 (1.34)	0.57	0.15–2.17	0.42				
**No. of Bedrooms (>2)**	14 (2.64)	1.51	0.48–4.78	0.39				
**Household trash (peridomicile)**	26 (2.00)	0.73	0.24–2.16	0.36	5 (0.38)	0.35	0.08–1.59	0.70
**VCP insecticide spraying (>12 months)**	47 (2.52)	1.88	0.34–10.33	0.32				
**Rocks/ bricks (peridomicile)**					2 (0.25)	0.31	0.05–1.78	0.63
**Firewood (peridomicile)**					11 (0.80)	3.03	0.53–17.18	0.57
**Bush or scrub (peridomicile)**					12 (0.71)	2.88	0.38–21.70	0.38

*Model_averaged effect-sizes (OR) from the final 8192-model set

**Model_averaged effect-sizes (OR) from the final 512-model set

Relative importance (RI) of variables was assessed by multi-model inference based on Akaike's information criterion

OR: Odds Ratio; 95% CI: Confidence Interval.

VCP: Vector control program Insecticide spraying

Vegetation type was a major determinant of peridomicile infestation by *T*. *carrioni* (w = 1.00) (Table 6). Households located in green low mountain forest had higher risk of infestation. Accumulation of household trash in peridomicile was a secondary factor in defining peridomicile (w = 0.7) infestation with a high protective effect. Variables that showed limited importance were: accumulation of rocks/bricks (w = 0.63) and firewood (w = 0.57) in the peridomicile, well drinking water (w = 0.52), self-reported insecticide spraying (w = 0.51), presence of bush or scrub (w = 0.38) and fruit trees (w = 0.37) in the peridomicile.

### Natural infection of triatomines by trypanosomes

Of the 754 triatomines examined, 10.6% were infected with *T*. *cruzi* and 1.7% were infected with *T*. *rangeli* (Table 7). All *T*. *cruzi* isolates that were genotyped belong to the DTU TcI.

**Table 7:** *Trypanosoma cruzi* and *T*. *rangeli* infection of triatomines collected in rural communities in Loja province.

	Domicile			Peridomicile			Total		
Triatomine	No. Triatomines analyzed	infected with *T*. *cruzi* (%)	infected with *T*. *rangeli* (%)	No. Triatomines analyzed	infected with *T*. *cruzi* (%)	infected with *T*. *rangeli* (%)	No. Triatomines analyzed	infected with *T*. *cruzi* (%)	infected with *T*. *rangeli* (%)
*R*. *ecuadoriensis*	74	22 (29,7)	2 (2,7)	260	26 (10,0)	3 (1,2)	334	48 (14,4)	5 (1,5)
*T*. *carrioni*	179	8 (4,5)	2 (1,1)	120	10 (8,3)	0 (0)	299	18 (6,0)	2 (0,7)
*P*. *chinai*	88	12 (13,6)	4 (4,5)	21	2 (9,5)	2 (9.5)	109	14 (12,8)	6 (5,5)
*P*. *rufotuberculatus*	12	0 (0)	0 (0)	0	------	------	12	0 (0)	0 (0)
**Total**	353	42 (11,9)	8 (2,3)	401	38 (9,5)	5 (1,2)	754	80 (10,6)	13 (1,7)

*Rhodnius (R)*, *Triatoma (T)*, *Pastrongylus (P)*

Three out of the four species of triatomine collected presented infection with *T*. *cruzi*. *R*. *ecuadoriensis* and *P*. *chinai* showed high rates of infection, 14.4% and 12.8%, respectively; while *T*. *carrioni* presented 6% of infection. No *Trypanosoma* sp. were detected in the 12 *P*. *rufotuberculatus* individuals that were examined.

Overall, higher infection rates with *T*. *cruzi* were detected in the domicile (11.9%) than in the peridomicile (9.5%). This tendency was evident especially in *R*. *ecuadoriensis* which showed three times higher infection in the domicile (29.7%) than in the peridomicile (10%). In *P*. *chinai* the infection rate in the domicile was 13.6% while 9.5% in the peridomicile. *T*. *carrioni* presented a different tendency in which the infection in the peridomicile (8.3%) was higher than in the domicile (4.5%) (Table 7).

Although less frequent, *T*. *rangeli* infection was detected in the three species in the domicile but only infecting *R*. *ecuadoriensis* and *P*. *chinai* in the peridomicile. Mixed infections with *T*. *cruzi* and *T*. *rangeli* were detected in all three species in rates <1%.

Among the 751 specimens tested using both methods, 42 (5.6%) were positive by microscopy for trypanosomatids (determined by the presence of flagellates in the thick smear of intestinal content) and 90 (12.0%) were positive (for the presence of *T*. *cruzi* and *T*. *rangeli*) by PCR (Table 8). The sensitivity of microscopy was 30.0% (IC 95% 20.8–40.6) and the specificity was 97.7% (IC 95% 96.3–98.7). The two methods had a poor degree of agreement (*κappa* = -0.3422, p > 0.05).

**Table 8:** Comparison of PCR and Microscopy for *T*. *cruzi* and *T*. *rangeli* detection in triatomines.

	PCR		
ME	Positive	Negative	Total
Positive	27	15	42
Negative	63	646	709
**Total**	90	661	751

ME: microscopy examination;

PCR: Polymerase Chain Reaction

## Discussion

This first comprehensive survey of triatomines in southern Ecuador found intradomiciliary and peridomiciliary infestation by *R*. *ecuadoriensis*, *T*. *carrioni*, *P*. *chinai*, and *P*. *rufotuberculatus* in all ecological zones below 2,200 masl in Loja Province. Triatomine infestation was characterized by high rates of colonization, indicating a well-adapted and thriving bug population. Triatomines were readily recognized by the population as a nuisance and inflammatory reactions to bites were recounted. Infestation was mainly associated with presence of domestic animals (guinea pigs and pigs), poor sanitary infrastructure, accumulation of agricultural products outdoors and presence of fruit trees outdoors. Houses located in the green low mountain forest and dry mountain forest had a higher risk to be infested. Vector control program insecticide spraying was a major protective factor. However, the fact that most variables studied have either none or weak association with triatomine infestation could be related to overall abundance of substandard living environments through rural areas of Loja province, where 93.2% of the population lives in chronic poverty otherwise defined as unmeet basic needs [11]. Triatomine infestation was higher in some counties of the province, specifically Calvas, Celica, Espíndola, Gonzanamá, Paltas, and Quilanga. Poverty among the population living in these counties ranges from 70 to 90%, some the highest in Loja Province and Ecuador[11]. Among infested communities, more than half had infestation rates higher than 10%; one-third had infestation rates higher than 20%. When coupled with the finding of a 10.6% rate of *T*. *cruzi* infection in collected triatomines, these findings indicate a high risk of transmission of *T*. *cruzi* in Loja Province.

The results of this study corroborate and expand on previous reports regarding triatomines in southern Ecuador [6,8,13]. Although triatomines were found in all ecological regions below 2,200 masl, there were clear ranges of altitude: *R*. *ecuadoriensis* was found below 1,800 masl and *T*. *carrioni* at altitudes above 800 masl. The fact that there was significant geographic overlap between species, especially *R*. *ecuadoriensis* and *P*. *chinai*, and *R*. *ecuadoriensis* and *T*. *carrioni* needs to be taken into consideration for the monitoring and evaluation of vector control interventions.

*R*. *ecuadoriensis* and *T*. *carrioni* were found more frequently than *P*. *chinai* and *P*. *rufotuberculatus*, leading us to conclude that while all four species can colonize domiciles, *R*. *ecuadoriensis* and *T*. *carrioni* are particularly adapted to do so. Previous reports have indicated that *P*. *chinai* and *P*. *rufotuberculatus* are primarily sylvatic [6],[22],[23]. However, our findings of colonization indexes of 63% for *P*. *chinai* and 58% for *P*. *rufotuberculatus*, plus populations with individuals of all nymphal instar stages, indicate domiciliation. The adaptation of *P*. *chinai* to reproduction in synanthropic habitats, coupled with a 12.8% *T*. *cruzi* infection rate, indicates that these species could become important vectors if they occupy the ecological niches that may be left open as a result of control interventions targeted toward other species.

While earlier reports [6],[12],[24] suggested that triatomines in Loja province are found only in domestic and peridomestic habitats, *T*. *cruzi* infected sylvatic *R*. *ecuadoriensis* has since been documented throughout the province [8,13]. To date we have not found any sylvatic *P*. *chinai* or *T*. *carrioni* in this region. Preliminary analyses indicate that the abundance of domestic/peridomestic infestation correlates with the abundance of sylvatic *R*. *ecuadoriensis*, which has important implications for control interventions in terms of reinfestation risk and early detection of this process. Our previous results suggest that there is flow of *R*. *ecuadoriensis* from the sylvatic to the domestic environments [25]. This is somewhat similar to what has been previously reported for *T*. *infestans* in Bolivia [26]. It will be interesting to further study the potential role of peridomestic structures as barriers for domestic infestation as previously proposed for *T*. *infestans* [27].

The presence of *T*. *rangeli* in this area has been previously reported [8,28]. In our study, a relatively low *T*. *rangeli* infection was observed in three of the four species of triatomines examined. The presence of this parasite is important for two reasons: First *T*. *rangeli* is widely believed to be pathogenic to triatomines, and thus might affect the survival and strength of bug populations. Second, transmission of this parasite via direct inoculation during feeding is more effective than *T*. *cruzi* transmission via contaminated feces. Although the persistence of the parasite in vertebrate blood appears to be transient, the presence of anti-*T*. *rangeli* antibodies might be a confounding factor for epidemiological studies [29].

In general, the detection of infection with trypanosomes (*T*. *cruzi* and *T*. *rangeli*) by microscopy was less sensitive than PCR. Moreover, the identification of the species of trypanosomes present in thick smears of intestinal content proved difficult and mixed infections were not detected by microscopy.

The high infestation rate found in our study is consistent with a recent report of 7.1% anti-*T*. *cruzi* seropositivity among school children <10 years of age found in Loja Province; in that study the rate of seropositivity was lower in older age groups, suggesting a fairly recent increase in transmission rates in this region [30]. Earlier studies had found seroprevalence in Loja Province to be 3.9% and 3.6% [6],[7]. Previous risk factor analysis for anti-*T*. *cruzi* seroprevalence in Ecuador includes data from Loja Province. The results show weak association of house construction materials, but not characteristics of the peridomicile, with anti-*T*. *cruzi* seropositivity [7],[30]. The power of analyses based on seropositivity is limited due to it being a cumulative measure of exposure to *T*. *cruzi*. In spite of the probable existence of congenital transmission in this area, the current analysis based on the presence of the vector is more appropriate to determine factors related to current *T*. *cruzi* transmission risk.

One of the major problems faced by field research surveys is quality control of the efforts. To address this problem, theoretical and practical training was provided to field personnel of the National Chagas Disease Program and the National Vector Control Services from many regions of the country. In addition, field teams were often accompanied by either Ecuadorian students and staff from Pontifical Catholic University of Ecuador and/or foreign students participating in Education Abroad programs organized by the Tropical Disease Institute at Ohio University.

Before the visit by our research team, knowledge about Chagas disease and its vectors was consistently low to nonexistent among the population in this region [6]. The process of searching for triatomines is highly disruptive to inhabitants of the domicile being examined. Besides the understandable resistance to allowing strangers to enter and search a house, the process is time consuming (at least 60 min). Moreover, if a domicile is found to be infested, it needs to be prepared with help from the inhabitants and then sprayed with insecticide (at least an additional 60 min). However, the educational efforts of this study, which employed interactive delivery of knowledge at each household and at schools, were well received. These community education efforts increased the acceptance of the entomological survey and produced an important commitment to community-based triatomine surveillance. Indeed, a pilot surveillance system was put in place in 34 communities in 2006, which during the next 12 months produced frequent reports of triatomine infestation and reinfestation. Regretfully, the program had to be discontinued in 2007 due to the lack of action and response to these reports by local vector control services. Nevertheless, this approach was proved to be feasible and effective at the community level and should be an integral part of future control efforts.

The results of this study probably would be extended to northern Peru, particularly in Piura province, which has similar ecological characteristics, human habitation, and vector species [31],[32]. Interestingly, a recent report by Alroy *et*. *al* [33] only found *P*. *lignarius* (formely *P*. *herreri*) in four communities located further south, in Cutervo Province in northern Peru. However, we did not find this species in our study in southern Ecuador. Current research by our group is aimed at understanding the *T*. *cruzi* transmission cycles in the southern region of Ecuador via examination of the genetic relationships among and between triatomine populations occupying domestic, peridomestic, and sylvatic habitats and the trypanosomes that infect vectors, reservoirs, and humans [19]. Future efforts will be aimed at increasing the capacity of the local health care system to detect, accurately diagnose, and treat acute and chronic Chagas disease[34].

### Overall implication for public health and control efforts

Chagas disease has historically been associated with poverty [35],[36]. The results of this study reinforce this notion. Estimating the actual risk of *T*. *cruzi* transmission in southern Ecuador would be very complex. However, the national census conducted in Ecuador in 2010 shows that Loja province has 155,046 domiciles of which 39% (60,420) are located in rural areas below 2,200 masl[11]. Therefore, taking into consideration only the population data of rural parishes below 2200 masl, about 155,533 people live in areas where triatomines are present. Moreover, an estimated 18,622 rural households have dirt floors, 24,562 have adobe walls and 13,536 dwellings lack any form of sanitation [10,11]. Consequently, approximately 98,248 people living in Loja province are exposed to a high risk of *T*. *cruzi* transmission due to an increased risk of triatomine infestation.

The situation is complicated by recent findings that indicate a high rate of triatomine reinfestation as early as six months year after insecticide is applied by spraying [37]. The high household reinfestation observed could be explained by the widespread presence of sylvatic *R*. *ecuadoriensis* in this region [13]. Morphometric analysis shows that the sylvatic populations of this species have high adaptive plasticity that allows them to readily establish colonies in houses and their surroundings [25].

Our findings support the conclusion that transmission of *T*. *cruzi* is actively occurring in Loja Province. Presently, government control efforts in this region are hampered by an ineffective local administration that had carried out minimal and uncoordinated triatomine control activities tangential to mosquito control. To date, the only substantial triatomine control efforts have been limited to the joint activities undertaken by our academic research group with support from the National Chagas Control Program. The results of this study provide important information to the National Chagas Control Program and the Ministry of Health whom should step up their vector surveillance and control activities in this region. Moreover, these results should prompt the implementation of a renewed effort to conduct seroprevalence studies and to provide this region with currently lacking Chagas disease diagnostic and treatment capacity.

A systematic, sustained, and monitored vector control intervention that incorporates community education and community-based surveillance, and that addresses the socio-economic structural causes of poverty with particular attention to substandard housing construction, needs to be implemented in southern Ecuador.

## Supporting Information

S1 Table Entomological indexes and altitude range of triatomine infestation in rural communities of Loja Province. (DOCX)

S2 Table Bivariate analysis of house and peridomestic characteristics, livestock and rodent/marsupial pests with triatomine infestation in rural communities of Loja Province. (DOCX)
