# Supplementary material for: Comprehensive Survey of Domiciliary Triatomine Species Capable of Transmitting Chagas Disease in Southern Ecuador
Source: PLoS Negl Trop Dis. 2015 Oct 6;9(10):e0004142. doi: 10.1371/journal.pntd.0004142 (PMC4595344; doi:10.1371/journal.pntd.0004142)
Supplement: S1 Table — (DOCX) [file pntd.0004142.s001.docx]

**Table S1. Entomological indexes and altitude range of triatomine infestation in rural communities of Loja Province.**

| Community^a^ | Domiciles  examined (no.) | Altitude min and max ^b^ | Triatomines  collected (no.) | Infestation Index (% colonization) ^c^ | | | | |
| --- | --- | --- | --- | --- | --- | --- | --- | --- |
|  |  |  |  | *R. ecuadoriensis* | *T. carrioni* | *P. chinai* | *P. rufotuberculatus* | *Total* |
| Calvas County |  |  |  |  |  |  |  |  |
| Chaquizhca | 31 | 991 - 1937 | 70 | 32.3 (40) |  | 18.6 (63) |  | 48.4 (53) |
| Guara | 22 | 1117 - 1307 | 593 | 22.7 (100) |  | 20.0 (100) |  | 36.4 (100) |
| Jacapo | 53 | 1635 - 1785 | 71 |  | 11.3 (83) |  |  | 11.3 (83) |
| Pindo | 40 | 1265 - 1359 | 18 | 7.5 (100) |  |  |  | 7.5 (100) |
| Suanamaca | 41 | 1862 - 1937 | 93 |  | 4.9 (100) |  |  | 4.9 (100) |
| Catamayo County |  |  |  |  |  |  |  |  |
| El Huayco | 34 | 1228 - 1413 | 233 | 5.9 (100) |  | 2.9 (0) |  | 8.8 (67) |
| La Extensa | 47 | 1244 - 1272 | 397 | 4.3 (100) |  | 8.5 (50) |  | 10.6 (60) |
| Zambi | 79 | 1444- 1489 | 13 |  | 3.8 (100) |  |  | 3.8 (100) |
| Celica County |  |  |  |  |  |  |  |  |
| Algarrobillo | 49 | 741 - 800 | 191 | 16.3 (75) |  |  | 2.0 (100) | 18.4 (78) |
| La Cienega | 42 | 810 -869 | 531 | 21.4 (78) |  |  | 7.1 (33) | 26.2 (64) |
| Chaguarpamba County | |  |  |  |  |  |  |  |
| Achiote | 25 | 1022 | 14 |  | 4.0 (100) |  |  | 4.0 (100) |
| Amarillos | 39 | 1179 - 1221 | 9 |  | 7.7 (67) |  |  | 7.7 (67) |
| Buenavista | 154 | 1150 | 3 | 0.6 (100) |  |  |  | 0.6 (100) |
| Jorupe | 26 | 1336 - 1397 | 142 |  | 11.5 (100) |  |  | 11.5 (100) |
| Venecia | 21 | 1371 - 1438 | 97 |  | 14.3 (100) |  |  | 14.3 (100) |
| Yaguachi | 45 | 831 - 1310 | 39 | 2.2 (100) | 13.3 (33) |  |  | 15.6 (43) |
| Espíndola County |  |  |  |  |  |  |  |  |
| Cangochara | 61 | 1762 - 2022 | 268 |  | 13.1 (88) | 1.6 (0) |  | 13.1 (88) |
| El Tambo | 56 | 1526 -1539 | 10 |  | 5.4 (100) |  |  | 5.4 (100) |
| El Tingo | 19 | 1440 - 1620 | 108 |  | 26.3 (80) |  |  | 26.3 (80) |
| Guarango | 18 | 1738 | 3 |  | 5.6 (0) |  |  | 5.6 (0) |
| Machay | 39 | 2040 - 2242 | 83 |  | 15.4 (100) |  |  | 15.4 (100) |
| Sanambay | 71 | 1758 - 2038 | 76 |  | 15.5 (73) |  |  | 15.5 (73) |
| Santa Teresita | 52 | 1839 - 1972 | 61 |  | 11.5 (83) |  |  | 11.5 (83) |
| Tundurama | 61 | 1584 - 2012 | 714 | 1.6 (0) | 21.3 (92) |  |  | 23.0 (86) |
| Gonzanamá County | |  |  |  |  |  |  |  |
| Chirimoyos | 19 | 1135 - 1255 | 11 | 5.3 (100) |  | 10.5 (100) |  | 15.8 (100) |
| Jurupe | 30 | 1622 - 1845 | 44 |  | 33.3 (60) |  |  | 33.3 (60) |
| San Jacinto | 11 | 1246 | 70 | 9.1 (100) |  |  |  | 9.1 (100) |
| Santa Ester | 27 | 1074 - 1206 | 45 | 18.5 (80) |  | 18.5 (80) |  | 29.6 (88) |
| Santa Rita | 20 | 1287 | 1 |  |  | 5.0 (0) |  | 5.0 (0) |
| Trigopamba | 42 | 1730 - 1848 | 12 | 2.4 (100) | 2.4 (100) |  |  | 4.8 (100) |
| Loja County |  |  |  |  |  |  |  |  |
| Comunidades | 30 | 1613 | 1 |  |  |  | 3.3 (0) | 3.3 (0) |
| Macará County |  |  |  |  |  |  |  |  |
| Laguar | 17 | 404 - 563 | 6 |  |  |  | 11.8 (50) | 11.8 (50) |
| Paltas County |  |  |  |  |  |  |  |  |
| Ashimingo | 29 | 869 - 1148 | 252 | 17.2 (80) |  | 3.4 (100) | 10.3 (67) | 24.1 (100) |
| Bramaderos | 61 | 867 - 1308 | 771 | 19.7 (100) |  | 1.6 (100) |  | 21.3 (100) |
| Coamine | 46 | 949 - 1368 | 1,479 | 10.9 (80) | 4.3 (100) | 15.2 (57) |  | 23.9 (73) |
| El Limon | 43 | 1028 - 1229 | 20 | 9.3 (50) |  | 4.7 (50) |  | 14.0 (50) |
| El Sauce | 28 | 1174 | 3 |  |  |  | 3.6 (100) | 3.6 (100) |
| Higuinda | 28 | 1328 | 230 | 3.6 (100) |  |  |  | 3.6 (100) |
| La Ramada | 10 | 949 - 961 | 40 | 20.0 (100) |  |  |  | 20.0 (100) |
| Lucarqui | 19 | 684 | 5 |  |  | 5.3 (100) |  | 5.3 (100) |
| Macandamine | 44 | 968 | 1 | 2.3 (0) |  |  |  | 2.3 (0) |
| Mogora | 15 | 905 | 9 | 6.7 (100) |  |  |  | 6.7 (100) |
| Naranjo | 53 | 902 - 904 | 68 | 3.8 (100) |  |  |  | 3.8 (100) |
| Naranjo Dulce | 37 | 1225 - 1601 | 49 | 10.8 (75) | 5.4 (50) | 5.4 (100) |  | 18.9 (86) |
| Sabanilla | 26 | 909 - 910 | 15 | 7.7 (50) |  |  |  | 7.7 (50) |
| Sacapianga | 17 | 1039 - 1107 | 400 | 35.3 (83) |  |  |  | 35.3 (83) |
| Tacoranga | 29 | 1136 - 1660 | 1,348 | 13.8 (100) | 3.4 (100) | 3.4 (100) |  | 17.2 (100) |
| Vega del Carmen | 40 | 1200 - 1365 | 124 | 5.0 (100) |  | 5.0 (0) |  | 7.5 (67) |
| Yamana | 77 | 1140 | 6 | 1.3 (100) |  |  |  | 1.3 (100) |
| Zapotepamba | 16 | 992 | 3 | 6.3 (100) |  |  |  | 6.3 (100) |
| Puyango County |  |  |  |  |  |  |  |  |
| Manguarquillo | 52 | 275 -281 | 41 | 5.8 (67) |  |  |  | 5.8 (67) |
| Pitayo | 21 | 675 | 3 |  |  |  | 4.8 (100) | 4.8 (100) |
| San Francisco | 13 | 837 - 861 | 226 | 15.4 (100) |  | 7.7 (100) |  | 23.1 (100) |
| Quilanga County |  |  |  |  |  |  |  |  |
| Galapagos | 34 | 1215 - 1393 | 733 | 29.4 (100) |  |  |  | 29.4 (100) |
| Jacapo-Quilanga | 22 | 1263 - 1558 | 73 |  | 18.2 (100) |  |  | 18.2 (100) |
| Santa Rosa | 39 | 1263 - 1712 | 220 | 15.4 (100) | 5.1 (100) |  |  | 20.5 (100) |
| Tuburo | 27 | 1219 - 1283 | 905 | 18.5 (100) |  |  |  | 18.5 (100) |
| Saraguro County |  |  |  |  |  |  |  |  |
| Chamical | 24 | 1388 | 1 |  |  | 4.2 (0) |  | 4.2 (0) |
| Sozoranga County |  |  |  |  |  |  |  |  |
| Gualguama | 9 | 1385 | 10 |  | 11.1 (100) |  |  | 11.1 (100) |
| Nueva Fátima | 38 | 1622-1645 | 22 | 5.3 (50) | 5.3 (100) |  |  | 10.5 (75) |
| Tumbunuma | 42 | 1662 - 1645 | 13 |  | 4.8 (100) |  |  | 4.8 (100) |
| Zapotillo |  |  |  |  |  |  |  |  |
| Jaguay Grande | 28 | 318 - 330 | 7 | 3.6 (0) |  | 7.1 (0) |  | 7.1 (0) |
| Limones | 61 | 175 | 11 |  |  | 3.3 (50) |  | 3.3 (50) |
| TOTAL | 3191 | 175-2242 | 11115 | 4.1 (83) | 3.4 (82) | 1.6 (63) | 0.4 (58) | 8.8 (80) |

^a^ Communities where no infested domiciles were found: Calvas County [Chingulle (39), Usaime (37)], Catamayo County [Algarrobera (27), Chapamarca (20), San Bernabe (42)], Celica County [Guineo (17)], Chaguarpamba County [Cucumate (21), Lozumbe (25), Mizhquillana (29)], Espíndola County [Potrerillos (11)], Loja County [Elvira (36), Palmira (81)], Macará County [Algodonal (31)], Olmedo County [La Delicia (37)], Paltas County [La Coordillera (19), Las Cochas (76), Palo Blanco (11), Playas (41)], Pindal County [Milagro (38), Misama (39)], Puyango County [Alahumbo (10), Guahinche (7), La Vega (16)], Saraguro County [Santa Rosa (5), Zapotepamba (7)], Sozoranga County [Chorora (14), Viviates (21)], Zapotillo County [Cochas de Almendro (46), Tronco Quemado (39)].

^b^ Minimum and maximum altitude of triatomine infested DUs in meters above sea level.

^c^ Infestation rate (100 x number of houses infested /number of houses searched) and colonization index (100 x number of houses with nymphs/number of houses infested) (WHO 2002).
