# Supplementary material for: Comprehensive Survey of Domiciliary Triatomine Species Capable of Transmitting Chagas Disease in Southern Ecuador
Source: PLoS Negl Trop Dis. 2015 Oct 6;9(10):e0004142. doi: 10.1371/journal.pntd.0004142 (PMC4595344; doi:10.1371/journal.pntd.0004142)
Supplement: S2 Table — (DOCX) [file pntd.0004142.s002.docx]

Table S2. Bivariate analysis of house and peridomestic characteristics, livestock and rodent/marsupial pests with triatomine infestation in rural communities of Loja Province.

|  | **N** | **%** | ***Rhodnius ecuadoriensis*** | | | | | | |  | ***Triatoma carrioni*** | | | | | | |
| --- | --- | --- | --- | --- | --- | --- | --- | --- | --- | --- | --- | --- | --- | --- | --- | --- | --- |
|  |  |  | **Intradomicile** | | |  | **Peridomicile** | | |  | **Intradomicile** | | |  | **Peridomicile** | | |
|  |  |  |  | **95% CI** | |  |  | **95% CI** | |  |  | **95% CI** | |  |  | **95% CI** | |
| **Factors** |  |  | **OR** | **Lower** | **Upper** |  | **OR** | **Lower** | **Upper** |  | **OR** | **Lower** | **Upper** |  | **OR** | **Lower** | **Upper** |
| **Surrogates Living Standard** | |  |  |  |  |  |  |  |  |  |  |  |  |  |  |  |  |
| Electricity | 2118 | 90.3 | 0.9 | 0.3 | 2.3 |  | 0.5 | 0.3 | 1.0 |  | 0.6 | 0.3 | 1.4 |  | 0.6 | 0.1 | 2.7 |
| Bedrooms > 2 | 2996 | 24 | 0.3 | 0.1 | 0.7 |  | 0.8 | 0.4 | 1.3 |  | 1.0 | 0.6 | 1.6 |  | 0.9 | 0.1 | 2.1 |
| Inhabitant crowding | 2104 | 53.4 | 1.7 | 0.9 | 3.1 |  | 1.3 | 0.8 | 2.2 |  | 1.3 | 0.7 | 2.4 |  | 0.7 | 0.3 | 2.2 |
| No latrine | 3017 | 41.3 | 2.5 | 1.5 | 4.2 |  | 2.1 | 1.3 | 3.3 |  | 2.4 | 1.5 | 3.8 |  | 1.3 | 0.6 | 2.5 |
| **Cooking fuel** |  |  |  |  |  |  |  |  |  |  |  |  |  |  |  |  |  |
| Natural gas ^b^ | 2120 | 78.5 | 0.6 | 0.3 | 1.0 |  | 1.0 | 0.5 | 1.8 |  | 0.3 | 0.2 | 0.5 |  | 0.0 | 0.0 | 0.2 |
| Firewood/coal ^b^ | 2119 | 81.2 | 1.1 | 0.5 | 2.4 |  | 2.1 | 0.9 | 4.9 |  | 5.6 | 1.3 | 23.1 |  | 0.8 | 0.8 | 0.8 |
| **Sewage** |  |  |  |  |  |  |  |  |  |  |  |  |  |  |  |  |  |
| Public sewer system | 2118 | 5.6 | 0.4 | 0.1 | 2.7 |  | 0.6 | 0.1 | 2.4 |  | - | - | - |  | - | - | - |
| Septic Tank | 2118 | 20 | 0.4 | 0.1 | 1.1 |  | 0.4 | 0.1 | 0.9 |  | - | - | - |  | - | - | - |
| Sewage to environment | 2118 | 60.4 | 2.1 | 1.1 | 4.2 |  | 2.0 | 1.1 | 3.5 |  | 7.6 | 2.7 | 21.2 |  | - | - | - |
| **Drinking water** |  |  |  |  |  |  |  |  |  |  |  |  |  |  |  |  |  |
| Piped water system | 2120 | 62.5 | 0.5 | 0.3 | 0.9 |  | 0.5 | 0.3 | 0.9 |  | 0.3 | 0.2 | 0.6 |  | 0.3 | 0.1 | 0.9 |
| Well | 2118 | 6.4 | 0.7 | 0.2 | 2.7 |  | 1.1 | 0.4 | 3.0 |  | 0.9 | 0.3 | 3.1 |  | 2.7 | 0.6 | 12.2 |
| Water from river or stream | 2118 | 31.6 | 2.2 | 1.2 | 4.0 |  | 1.9 | 1.1 | 3.1 |  | 3.2 | 1.8 | 5.8 |  | 2.5 | 0.9 | 7.6 |
| **Roof material** |  |  |  |  |  |  |  |  |  |  |  |  |  |  |  |  |  |
| Cement/asbestos/zinc | 3039 | 13.9 | 0.3 | 0.1 | 1.0 |  | 0.7 | 0.3 | 1.5 |  | 0.1 | 0.0 | 0.6 |  | 0.4 | 0.1 | 1.6 |
| Tile | 3039 | 85.4 | 3.5 | 1.1 | 11.3 |  | 1.5 | 0.7 | 3.2 |  | 4.8 | 1.5 | 15.2 |  | 1.8 | 0.5 | 5.8 |
| Palm, other | 3039 | 0.7 | - | - | - |  | - | - | - |  | 1.8 | 0.2 | 13.9 |  | 4.8 | 0.6 | 36.6 |
| **Wall material** |  |  |  |  |  |  |  |  |  |  |  |  |  |  |  |  |  |
| Cement/brick | 3018 | 26 | 0.5 | 0.3 | 1.0 |  | 0.6 | 0.3 | 1.0 |  | 0.3 | 0.1 | 0.6 |  | 0.6 | 0.3 | 1.5 |
| Adobe | 3018 | 70.2 | 1.8 | 1.0 | 3.4 |  | 1.6 | 0.9 | 2.9 |  | 3.3 | 1.7 | 6.3 |  | 1.6 | 0.7 | 3.8 |
| Wood | 3018 | 1.5 | 1.1 | 0.1 | 8.1 |  | - | - | - |  | - | - | - |  | - | - | - |
| Cane, other | 3018 | 2.6 | 0.6 | 0.1 | 4.5 |  | 1.6 | 0.5 | 5.1 |  | 1.4 | 0.4 | 4.6 |  | 1.2 | 0.2 | 8.6 |
| **Floor** |  |  |  |  |  |  |  |  |  |  |  |  |  |  |  |  |  |
| Cement/tile/wooden parquet | 3032 | 35.9 | 1.0 | 0.6 | 1.7 |  | 0.8 | 0.5 | 1.3 |  | 0.2 | 0.1 | 0.4 |  | 0.2 | 0.1 | 0.6 |
| Wood boards | 3032 | 17.2 | 0.4 | 0.2 | 1.0 |  | 0.7 | 0.4 | 1.4 |  | - | - | - |  | 0.5 | 0.1 | 1.5 |
| Cane, other | 3032 | 1.2 | - | - | - |  | - | - | - |  | - | - | - |  | - | - | - |
| Dirt | 3032 | 58.1 | 1.7 | 1.0 | 2.9 |  | 2.3 | 1.4 | 3.8 |  | 3.5 | 2.0 | 6.1 |  | 3.4 | 1.4 | 8.2 |
| **Intradomicile storage** |  |  |  |  |  |  |  |  |  |  |  |  |  |  |  |  |  |
| Firewood ^b^ | 2102 | 15.8 | 1.1 | 0.5 | 2.4 |  | 1.0 | 0.5 | 2.0 |  | 1.4 | 0.7 | 2.8 |  | 1.8 | 0.5 | 6.6 |
| Agricultural products ^b^ | 2099 | 60.1 | 2.1 | 1.0 | 4.1 |  | 1.8 | 1.0 | 3.2 |  | 1.5 | 0.8 | 2.8 |  | 0.9 | 0.3 | 2.9 |
| **Previous Spraying** |  |  |  |  |  |  |  |  |  |  |  |  |  |  |  |  |  |
| Sprayed < 12 months | 3191 | 14.5 | 0.5 | 0.2 | 1.2 |  | 0.1 | 0.0 | 0.6 |  | 0.1 | 0.0 | 0.5 |  | - | - | - |
| **Livestock** |  |  |  |  |  |  |  |  |  |  |  |  |  |  |  |  |  |
| Chickens, other birds | 3029 | 90.5 | 7.0 | 1.0 | 50.6 |  | - | - | - |  | 1.7 | 0.7 | 4.3 |  | 3.5 | 0.5 | 25.8 |
| Dogs | 3032 | 80.5 | 1.0 | 0.5 | 1.8 |  | 3.6 | 1.4 | 8.8 |  | 1.5 | 0.8 | 2.8 |  | 1.9 | 0.7 | 5.4 |
| Guinea pigs (indoor/outdoor) | 3028 | 25.1 | 1.2 | 0.7 | 2.1 |  | 0.5 | 0.3 | 1.0 |  | 2.9 | 1.9 | 4.5 |  | 1.8 | 0.9 | 3.6 |
| Guinnea Pig Pen (outdoor) | 753 | 82.7 | 0.3 | 0.1 | 0.9 |  | 0.7 | 0.3 | 18.1 |  | 0.4 | 0.2 | 0.8 |  | 1.2 | 0.3 | 5.3 |
| Pigs | 3031 | 64.1 | 3.6 | 1.8 | 7.3 |  | 3.1 | 1.7 | 5.9 |  | 1.4 | 0.9 | 2.2 |  | 1.1 | 0.5 | 2.2 |
| Sheep or goats | 3029 | 19.4 | 2.4 | 1.5 | 4.0 |  | 2.0 | 1.3 | 3.3 |  | 0.5 | 0.3 | 1.1 |  | 0.7 | 0.3 | 1.8 |
| Cats | 3015 | 56.9 | 1.3 | 0.8 | 2.2 |  | 1.8 | 1.1 | 2.9 |  | 1.3 | 0.8 | 2.1 |  | 0.8 | 0.4 | 1.6 |
| **Peridomicile** |  |  |  |  |  |  |  |  |  |  |  |  |  |  |  |  |  |
| Firewood | 2097 | 64.6 | 1.3 | 0.7 | 2.4 |  | 1.9 | 1.0 | 3.6 |  | 1.4 | 0.7 | 2.6 |  | 3.0 | 0.7 | 13.7 |
| Wood for construction | 2087 | 20.2 | 1.0 | 0.5 | 2.1 |  | 1.3 | 0.7 | 2.4 |  | 0.8 | 0.4 | 1.6 |  | - | - | - |
| Rocks/brick piles | 2087 | 38.3 | 0.8 | 0.4 | 1.5 |  | 0.8 | 0.5 | 1.4 |  | 0.7 | 0.4 | 1.3 |  | 0.3 | 0.1 | 1.3 |
| Household trash | 2085 | 60.7 | 0.1 | 0.7 | 2.5 |  | 1.5 | 0.8 | 2.6 |  | 0.7 | 0.4 | 1.3 |  | 0.4 | 0.1 | 1.2 |
| Agricultural refuse | 2091 | 31.2 | 1.6 | 0.9 | 3.0 |  | 1.7 | 1.0 | 2.9 |  | 0.9 | 0.5 | 1.6 |  | 1.0 | 0.3 | 3.2 |
| Agricultural products | 2089 | 25.6 | 0.9 | 0.5 | 1.8 |  | 2.1 | 1.2 | 3.6 |  | 1.1 | 0.6 | 2.0 |  | 1.3 | 0.4 | 4.2 |
| Bushes (arbustos) | 2097 | 85.9 | 1.3 | 0.5 | 3.4 |  | 1.2 | 0.5 | 2.6 |  | 1.9 | 0.7 | 5.2 |  | 2.0 | 0.3 | 15.2 |
| Fruit trees | 2088 | 58.2 | 0.5 | 0.3 | 0.9 |  | 1.1 | 0.7 | 2.0 |  | 2.3 | 1.2 | 4.3 |  | 2.4 | 0.7 | 8.8 |
| Brush or scrub | 2090 | 80 | 0.2 | 0.7 | 4.0 |  | 0.9 | 0.5 | 1.8 |  | 1.2 | 0.6 | 2.6 |  | 3.0 | 0.4 | 23.3 |
| Palm trees <30 m | 2078 | 3.7 | 1.2 | 0.2 | 5.2 |  | 1.5 | 0.4 | 4.8 |  | - | - | - |  | - | - | - |
| **Pests** |  |  |  |  |  |  |  |  |  |  |  |  |  |  |  |  |  |
| Mice | 2100 | 37.2 | 1.0 | 0.6 | 1.9 |  | 1.2 | 0.7 | 2.1 |  | 1.2 | 0.7 | 2.2 |  | 1.4 | 0.5 | 4.3 |
| Rats | 2099 | 39.9 | 1.1 | 0.6 | 2.0 |  | 0.8 | 0.4 | 1.3 |  | 3.4 | 1.8 | 6.3 |  | 5.1 | 1.4 | 18.5 |
| Opposum | 2097 | 13.6 | 1.0 | 0.4 | 2.4 |  | 1.3 | 0.7 | 2.0 |  | 1.1 | 0.5 | 2.4 |  | 1.2 | 0.3 | 5.2 |
